# Steatotic liver disease and liver transplantation: Candidate selection and post-transplant management

**DOI:** 10.1016/j.jhepr.2025.101704

**Published:** 2025-12-02

**Authors:** Jordi Colmenero, Gonzalo Crespo, Line Carolle Ntandja Wandji, Yiliam Fundora, Alexandre Louvet

**Affiliations:** 1 Liver Unit, Hospital Clínic, Barcelona, Spain; 2 Surgery Department, Hospital Clínic, Barcelona, Spain; 3 Institut de Investigacions Biomèdiques August Pi-i-Sunyer (IDIBAPS), CIBERehd (Centro de Investigación Biomédica en Red), University of Barcelona, Spain; 4 Service des Maladies de l’Appareil Digestif, Hôpital Huriez, Lille cedex France

**Keywords:** MASLD, MASH, MetALD, ALD, alcohol-related liver disease, metabolic-associated liver disease, disease recurrence, management, biomarkers

## Abstract

Steatotic liver disease (SLD), which encompasses alcohol-related liver disease (ALD), metabolic dysfunction-associated steatotic liver disease (MASLD), and MASLD with increased alcohol intake (MetALD), is now the leading cause of liver transplantation (LT) worldwide. ALD and MASLD have become the first and second leading indications for LT (41% and 12% in Europe, respectively), with MetALD accounting for a rapidly increasing proportion of transplants (8-10%). Candidate evaluation must be multidisciplinary and account for the complex interplay between alcohol use, metabolic syndrome, cancer, cardiovascular disease, and obesity. Early LT for severe alcohol-related hepatitis is an established option in selected patients after the evaluation of alcohol use disorder (AUD) by addiction specialists. While the duration of abstinence remains a predictor of post-LT alcohol relapse in ALD recipients, an integrative assessment is required, and prolonged abstinence is no longer an absolute prerequisite. Cardiovascular risk stratification and assessment of frailty and metabolic comorbidities are essential. Obesity management includes lifestyle interventions, pharmacotherapy, and bariatric surgery in selected cases. SLD recipients generally demonstrate good 5-year survival (>75%), but long-term outcomes are influenced by cardiovascular events, malignancies, and alcohol relapse, with survival falling below 65% at 10 years. Early detection and management of alcohol relapse after LT are critical to optimising long-term outcomes. MASLD recurrence is common, but its impact on graft survival appears modest. Management focuses on controlling cardiometabolic risk factors, with emerging roles for GLP-1 receptor agonists and multidisciplinary care. Donor SLD is a growing concern. Normothermic and hypothermic oxygenated perfusion substantially expand donor utilisation – with up to ∼70% of marginal or previously discarded grafts now salvaged – and improve graft viability by reducing early allograft dysfunction by 60%. Further research is needed to refine risk stratification, develop effective pharmacotherapies, and optimise perfusion protocols for steatotic grafts.

## Graphical abstract

**Figure:**
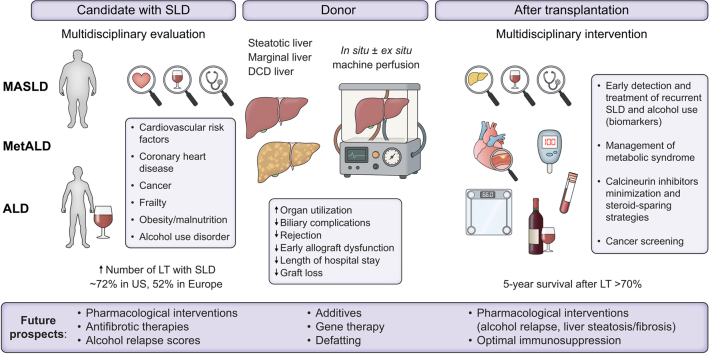

**Keypoints**

• The number of LT candidates with SLD and donors with steatotic grafts is rising worldwide.
• The selection of LT candidates with SLD requires a multidisciplinary approach (hepatology, surgery, addiction, endocrinology/nutrition, social care) to evaluate alcohol use, cancer risk, metabolic status, and cardiovascular comorbidities.
• LT recipients with SLD face increased risks of metabolic complications (obesity, diabetes, dyslipidaemia, hypertension), alcohol relapse, graft injury (steatosis, steatohepatitis, fibrosis), and long-term cardiovascular disease and cancer.
• Early detection of alcohol relapse, *de novo* MASLD or ALD, cardiometabolic risk factors, and cancer is critical and should be carried out using interviews, biomarkers, imaging, elastography and screening protocols.
• Personalised immunosuppression strategies, such as CNI minimisation and steroid avoidance, may help mitigate the risk of MASLD after LT.
• Targeted interventions such as GLP-1 receptor agonists, SGLT2 inhibitors, bariatric surgery, and comprehensive therapies for cardiometabolic risk factors and alcohol use disorder should be delivered through a multidisciplinary approach to optimise long-term outcomes.
• *Ex situ* machine perfusion techniques allow for the utilisation of marginal liver donors, decreasing the risk of biliary complications, early allograft dysfunction and graft loss.

## Definition of SLD

**Fig. 1:**
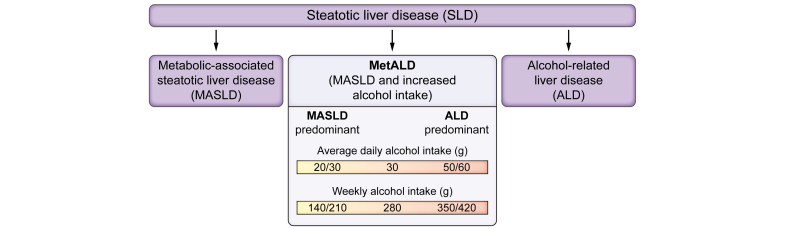
Main types of steatotic liver disease. ALD, alcohol-related liver disease; MASLD, metabolic dysfunction-associated steatotic liver disease; MetALD, MASLD with increased alcohol intake.

Steatotic liver diseases (SLD) include the whole spectrum of chronic liver diseases with steatosis of the liver, diagnosed histologically or by imaging. The disease spectrum ranges from simple steatosis to steatohepatitis with fibrosis and ultimately cirrhosis with end-stage liver disease and/or hepatocellular carcinoma (HCC). The current nomenclature classifies patients with steatosis into three main groups:^1^ metabolic dysfunction-associated steatotic liver disease (MASLD) – formerly termed non-alcoholic fatty liver disease, a label associated with stigma – alcohol-related liver disease (ALD) and MASLD with increased alcohol intake (MetALD) (Fig. 1). The distinction between MASLD, MetALD, and ALD is based on alcohol intake cut-offs of 20–30 g/day and 50–60 g/day, and it may be challenging given lifetime changes in body weight and alcohol consumption. Up to 17% of individuals categorised as having MASLD have a prior history of, or will be diagnosed with, AUD during follow-up.^2^^,^^3^ While cirrhosis is unlikely at alcohol intakes below 20–30 g/day,^4^ the relevance of the higher threshold (50-60 g/day) is debated^5^ and a recent Spanish study found that in individuals consuming >4 alcoholic drinks/week, liver fibrosis risk was driven by alcohol intake not cardiometabolic risk factors (CMRFs).^6^

## Epidemiology of SLD

SLD due to excessive alcohol consumption and metabolic dysfunction represents the most frequent cause of altered liver tests worldwide and the most important cause of end-stage liver disease.^7^ Chronic alcohol consumption and metabolic syndrome (MS) are prevalent in many countries, often coexist in a substantial proportion of individuals, and interact in a complex manner, as their effects are not simply additive but rather multiplicative.^8^^,^^9^ Biomarkers like phosphatidylethanol (PEth) can help clarify intake but should be viewed as supportive tools due to their limited detection window. Despite limitations, the new nomenclature highlights the overlap between alcohol use and metabolic dysfunction.^5^ Careful assessment of alcohol use is essential in MASLD to establish the diagnosis, guide management, and determine prognosis.

Globally, ALD affects 3.5% of the general population, 26.0% of hazardous drinkers, and 55.1% of individuals with AUD.^10^ Europe reports the highest per capita alcohol consumption worldwide,^11^ and ALD results in mortality at a younger age than metabolic dysfunction.^7^ MASLD and MetALD are the second and third most common liver diseases globally, driven by the rising prevalence of obesity, diabetes, and alcohol use. In Europeans aged 50–65, obesity and diabetes prevalence are 15–35% and 5–15%, respectively, with socioeconomic disparities.^7^ MASLD affects ∼32% in the Middle East, 30% in South America, 27% in Asia, and 24% in North America and Europe,^12^ with increasing rates among Hispanic and Asian populations.^13^ MetALD prevalence varies (2.1–8.3%) and accounts for 2–18% of SLD cases, especially in Western and Asian countries.^14^ Modelling predicts a growing burden of MASLD over the next 30 years, highlighting the need for proactive prevention..^15^ Both MASLD and ALD carry considerable stigma, largely driven by the perception that these conditions are self-inflicted. This perception, in turn, fosters the belief that patients “do not take care of themselves” and therefore do not deserve appropriate medical care. This moral judgment, based on stereotypes, causes social devaluation and isolation, which ultimately worsens healthcare disparities and limits access to treatment. The COVID-19 pandemic made these issues even worse by exposing a healthcare system with limited resources and greater access barriers. This further disadvantaged vulnerable patients and highlights the urgent need for a more equitable and holistic healthcare approach.

**Fig. 2:**
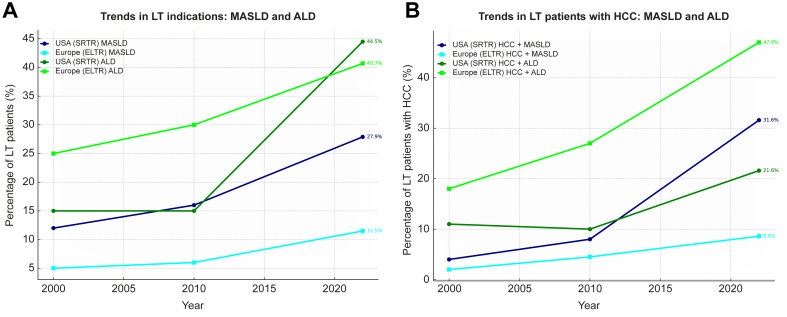
Trends in LT indications for MASLD and ALD in the US and Europe. (A) Proportion of LT recipients with MASLD and ALD. (B) Proportion of LT recipients with HCC with MASLD and ALD as the underlying disease. Data from ELTR and SRTR. ALD, alcohol-related liver disease; ELTR, European Liver Transplant Registry; HCC, hepatocellular carcinoma; LT, liver transplantation; MASLD, metabolic dysfunction-associated steatotic liver disease; SRTR, Scientific Registry of Transplant Recipients.

Liver-related mortality is highest in countries with high alcohol consumption and high prevalence of obesity,^7^ with important consequences on the need for liver transplantation (LT). ALD (with or without HCC) has become the primary indication for LT in Europe^16^ and Northern America.^17^ MASLD is now the second leading indication for LT in most Western countries^18^^,^^19^ and MetALD ranks as the third most common aetiology of liver disease among LT candidates in the US^20^ (Fig. 2A). These conditions significantly contribute to the burden of HCC,^21^^,^^22^ with a stepwise increase in risk observed from MASLD to MetALD and ultimately to ALD.^23^ Consequently, among LT recipients with HCC, the percentage of patients with underlying MASLD and ALD has markedly increased in the past two decades in the US and Europe^24^ (Fig. 2B).

## Selection of LT candidates with SLD

### ALD

Selection of candidates for LT is challenging in patients with ALD because of the complex relationship between improvement in liver function and abstinence. Originally, patients with ALD were not considered for LT if they had been abstinent for less than 6 months (the so-called “6-month” rule). A minimum abstinence period before waitlisting aims to identify patients who may recover without LT and those at lower risk of alcohol relapse after LT. It is generally accepted that liver disease improvement after abstinence occurs within the first 3-6 months, and liver recompensation may take 12-24 months.^25^ Recent studies have clarified the complex relationship between alcohol use and transplant need in ALD. A large cohort study (>80,000 patients) showed higher delisting rates for improvement in ALD compared to other aetiologies; however, this difference was not observed in those transplanted within 6 months, suggesting that abstinence benefits mainly those with less severe disease.^26^ A Spanish study (n = 420) found a 9% delisting rate for improvement and 13% for worsening or death, with lower MELD (model for end-stage liver disease) scores predicting improvement.^27^ Still, no MELD cut-off has been validated to support a wait-and-see approach.

Because alcohol relapse after LT can be associated with graft loss and increased mortality, selecting patients with ALD for LT is particularly relevant. A multidisciplinary evaluation, including addiction specialists, is essential before LT. A prolonged abstinence period before LT is linked to a lower risk of post-LT relapse.^28^ However, it must be emphasised that accurately predicting which recipients will remain abstinent after LT remains nearly impossible. For this reason, the rigid “6-month rule” for alcohol abstinence prior to LT has been abandoned by many centres and organisations,^29^^,^^30^ and the EASL guidelines in 2018 stated that patient selection should not be based on the 6-month rule alone.^31^ Many centres now accept patients with only a few months of sobriety – or even less in the case of severe alcohol-related hepatitis (SAH). The requirement for all liver transplant candidates to complete the same fixed period of abstinence has been superseded by a multidisciplinary specialist evaluation. This evaluation encompasses the patient’s suitability for transplantation, the risk of death without LT, and the need for proactive post-transplant care for AUD, including early relapse monitoring and engagement in addiction treatment. This strategy has been associated with comparable survival outcomes to traditional practices in ALD.^28^^,^^32^^,^^33^ In addition to short abstinence duration, several other factors are associated with relapse risk, and some have been incorporated into predictive scoring systems, including those endorsed by EASL^16^^,^^34^^,^^35^ (Box 1). Importantly, none of these individual factors constitutes an absolute contraindication to LT.^30^ Likewise, the prognostic performance of these tools is limited, with AUC values for relapse prediction not exceeding 0.80–0.85. In complex cases, ongoing and repeated engagement with the patient and their relatives is essential. Notably, most transplant committees lack formalised selection policies, and a prospective study in US LT centres revealed inconsistencies in candidate assessment, with ALD cases presenting the most frequent dilemmas.^36^

Box 1 **Factors associated with alcohol relapse after LT in patients with ALD.**

**Figure:**
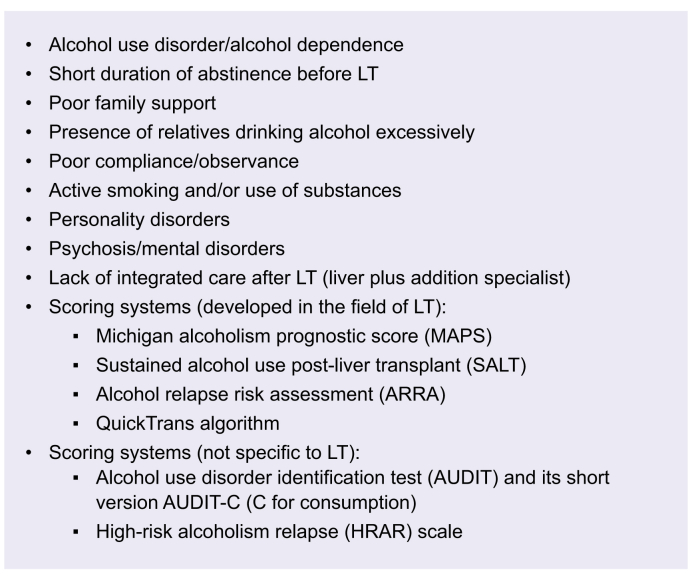

Alt-text: Box 1

ALD, alcohol-related liver disease; LT, liver transplantation.

#### Severe alcohol-related hepatitis (SAH)

Among patients with ALD, patients with SAH not responding to medical treatment represent a unique situation. By definition, these patients are not abstinent at the time of admission. In patients who do not respond to corticosteroids (defined by a Lille score ≥0.45),^37^ the risk of mortality at 6 months ranges from approximately 70% to 80%, with most deaths occurring within the first 2 months. Since no medical treatment has been proven effective in these non-responders, they face a therapeutic dead end. Since the first French and Belgian pilot study on early LT for patients with SAH who did not respond to steroids,^38^ several multicentre studies have consistently shown that early LT offers excellent 5-year patient survival in this population^39^ (Table 1). In most studies, the absence of an initial decompensation event was a prerequisite for listing a patient for LT and the waitlist decisions were made by a multidisciplinary team including nurses, residents, addiction specialists, surgeons, anaesthesiologists, and hepatologists. Building on this, the prospective QuickTrans programme^40^ developed a structured selection algorithm that integrates psychiatric and addiction assessments with liver team evaluation.

**Table 1:** Outcomes after early liver transplantation in severe alcohol-related hepatitis and alcohol-related liver disease.

First author (year)	Period	Study type	N	Setting	Patient survival	Alcohol relapse
Mathurin *et al.,* 2011^38^	2004–2010	Prospective	26	7 centers (BE, FR)	6-month: 77%	11.5% at 6 months
Im *et al.,* 2016^41^	2006–2012	Retrospective	9	US (1 center)	6-month: 89%	22% any; 11% severe
Lee *et al.,* 2018^42^	2006–2017	Prospective	147	17 US centers	1-year: 94%; 3-year: 84%	25% relapse at 3 years
Weeks *et al.,* 2018^43^	2012-2017	Retrospective	46	US (1 center)	6-month: 98%	28% at 1 year; 40% at 3 years
Sundaram *et al.,* 2018^44^	2015-2018	Retrospective	10	US (1 center)	1-year: 100%	10%: 1 year; 15% at 3 years
Lombardo-Quezada *et al.,* 2019^28^†	2004-2016	Retrospective	60	Spain (1 center)	5-year: 82%	17.6% severe at 3 years
Herrick-Reynolds *et al.,* 2021^32^†	2012–2018	Retrospective	163	US (1 center)	1-year: 94%	28%
Cotter *et al.,* 2021^45^	2012 -2017	Retrospective	46	US (1 center)	1-year: 93%; 5-year: 85%	45%; 15% severe
Lee *et al.,* 2022^46^	2006–2018	Retrospective	153	11 US centers	1-year: 95%; 5-year: 82%	29.4% (3-year median)
Louvet *et al.,* 2022^40^	2012–2016	Prospective	68	19 hospitals (FR, BE)	2-year: 90%	34% at 2 years
Weinberg *et al.,* 2022^47^∗	2007-2020	Retrospective	241	US multicenter	1-year: 86%; 2-year: 78%∗	22%: 1 year; 33%: 2-years∗
Germani *et al.,* 2022^48^	2013–2019	Retrospective	16	Italy (1 center)	2-year: 100%	12.5% at 6 months
Carrique *et al.,* 2021^33^†	2018-2020	Prospective	44	Canada (1 center)	2-year: 89%	6.8% (260 days mean)
Kulkarni *et al.,* 2023^49^	2020–2021	Retrospective	25	India ((1 center)	72% (median 551 days)	20% (87 days median)

∗ Outcomes of the 31 patients who underwent liver transplantation due to severe alcohol-related hepatitis with prior liver decompensation.

† Patients with alcohol-related liver disease with less than 6 months of abstinence.

A key component of the evaluation of ALD candidates, with or without SAH, is the somatic assessment. Because of alcohol exposure, patients with ALD are at an increased risk of aerodigestive cancers, breast cancer and ischaemic heart disease that correlates with number of standard drinks consumed daily.^50^ Moreover, the risk of lung cancer is higher in smokers, a common feature in patients with ALD. Even if there is no firm consensus on a specific pre-transplantation evaluation in candidates with ALD, it seems reasonable to check carefully for ear-nose-throat and oesophageal malignancies or preneoplastic lesions. Importantly, the 2015 and 2024 EASL guidelines on LT underlined the need for a systematic work-up for malignancies but does not recommend specific evaluation^16^^,^^51^ (Table 2). In contrast to cancer screening, the preoperative cardiovascular evaluation has been more extensively studied and EASL recommends systematic electrocardiogram and transthoracic echocardiography.^16^ This work-up must be adapted to the presence or not of cardiovascular risk factors, especially hypertension, diabetes and tobacco use, which are often present in patients with ALD.^5^^,^^52^

**Table 2:** Work-up for LT candidates with MASLD, MetALD or ALD.

	Tools	Action
**General (MASLD, MetALD & ALD)**		
Liver disease severity	MELD-Na, MELD 3.0	Definition of severity of alcohol-related hepatitis; Waiting list prioritisation
Screening for cancer	Hepatocellular carcinoma Population screening	Imaging of the liver (CT/MR) According to age and country
Frailty and sarcopenia	Liver frailty index, 6-minute walking test, CT assessment of sarcopenia	Multimodal prehabilitation Protein supplementation; late snacks.
Obesity/nutritional status	BMI, dietician referral	Diet. Bariatric surgery Child-Pugh A compensated patients with BMI >35
Cardiopulmonary assessment	ECG, echocardiogram, computed tomography coronary angiography (CTCA) with coronary artery calcification score (CACS) or invasive angiogram depending on risk factors and/or CAD-LT score	Rule out/treat cardiopulmonary disorders (porto-pulmonary hypertension, ischaemic heart disease). Pharmacological and non-pharmacological interventions
Renal function	Estimated glomerular filtration rate, proteinuria, hematuria (IgA glomerulopathy), renal biopsy	Consider simultaneous liver kidney transplantation (renal prognosis based on estimated glomerular filtration rate, chronicity, proteinuria, duration of acute kidney injury)
Psychosocial assessment	Social support, adherence, psychiatric evaluation	Social and pharmacological and non-pharmacological interventions
Smoking and substance use (cannabis, opioids, etc.)	Referral to addiction specialist	Tobacco cessation programme, treatment of use of cannabis or other substances including prescription drugs

**ALD-specific (includes patients with MetALD)**		
Evaluation of alcohol use	Addiction specialist	Pharmacological and non-pharmacological interventions
Biomarkers	EtG (urine/hair), PEth (blood)	Regular assessment
Alcohol-use disorders scores	AUDIT-C, SALT, ARRA, HRAR scores	To be performed by the addiction team

**MASLD-specific (includes patients with MetALD)**		
Metabolic syndrome	Blood pressure, HbA1c, lipidic profile, C-peptide	Multidisciplinary management, prevention

ALD, alcohol-related liver disease; CAD, coronary artery disease; EtG, ethyl glucuronide; MASLD, metabolic dysfunction-associated steatotic liver disease; MELD, model for end-stage liver disease; MetALD, MASLD with increased alcohol intake; PEth, phosphatidylethanol.

It should be noted that the pre-transplant work-up must be expedited in patients with severe liver dysfunction, particularly those with alcohol-related hepatitis or acute-on-chronic liver failure (ACLF), whether related to SAH or not. Since some patients with ALD will develop ACLF-2 or -3, and many patients with SAH meet ACLF-2/3 criteria, careful medical selection in the intensive care unit is essential to avoid transplanting patients who are too sick, particularly those with respiratory failure or severe sepsis.^53^ The concept of a transplantation window in the sickest patients underlines the need for a quick selection process.^54^ This is especially true in patients with SAH, in whom evidence of infection is present in at least 30% of patients.^55^ In patients with SAH and ACLF-3, three key considerations should be emphasised. First, patient selection criteria do not differ from those used in other aetiologies of liver disease. Second, futility criteria for LT in ACLF-3 are not fully validated and current guidelines recommend making decisions on a case-by-case basis, guided by independent predictors of post-transplant mortality. Third, although no single factor can be considered an absolute ‘no-go,’ several variables are consistently associated with poor post-LT outcomes: advanced age, frailty, uncontrolled sepsis (<24 h), active gastrointestinal bleeding, haemodynamic instability requiring high-dose norepinephrine, elevated arterial lactate (>9 mmol/L), respiratory failure (PaO_2_/FiO_2_ <150), a TAM (transplantation for ACLF-3 model) score >2, worsening clinical course, and complex surgical conditions (*e.g*. extensive portal vein thrombosis or prior LT).^56^ It should also be acknowledged that a substantial proportion of patients selected for early LT in the context of SAH will nevertheless die before LT, as illustrated by the QuickTrans study, in which 21 of 102 candidates succumbed while on the waitlist.^40^

### MASLD

Pretransplant evaluation of patients with cirrhosis due to MASLD or MetALD requires a multidisciplinary, individualised approach. Besides the general assessment for any patient with end-stage liver disease, all candidates should undergo thorough screening of MS-related complications, with particular focus on cardiovascular assessment^16^ and cancer (Table 2). Alcohol use must be thoroughly evaluated in patients with MASLD, as it is often underrecognised and associated with increased risk of adverse liver outcomes.^2^ Psychosocial evaluation and assessment of alcohol use and relapse risk are essential in patients with MetALD.^16^ Notably, 6–8% of LT candidates with MASLD have a history of bariatric surgery, predominantly sleeve gastrectomy, and these patients have increased risk of AUD and reduced transplant-free survival.^57^ Sarcopenia and frailty must also be assessed since both conditions are common in patients with MASLD^58^ and linked to higher post-LT mortality, particularly in acutely ill patients.^59^ Nutritional and functional prehabilitation is safe and improves frailty metrics in LT candidates yet the effect on mortality before and after LT requires further investigation.^60^ MS is frequent in LT candidates with SLD, particularly those with MASLD and MetALD who, by definition, also present with CMRFs.^61^ Among MS-related comorbidities, cardiovascular risk – particularly silent coronary artery disease (CAD) – and obesity are the factors that most strongly influence outcomes after LT. MASLD is also associated with a higher risk of solid and haematological cancer compared to the general population.^62^

#### Obesity

**Fig. 3:**
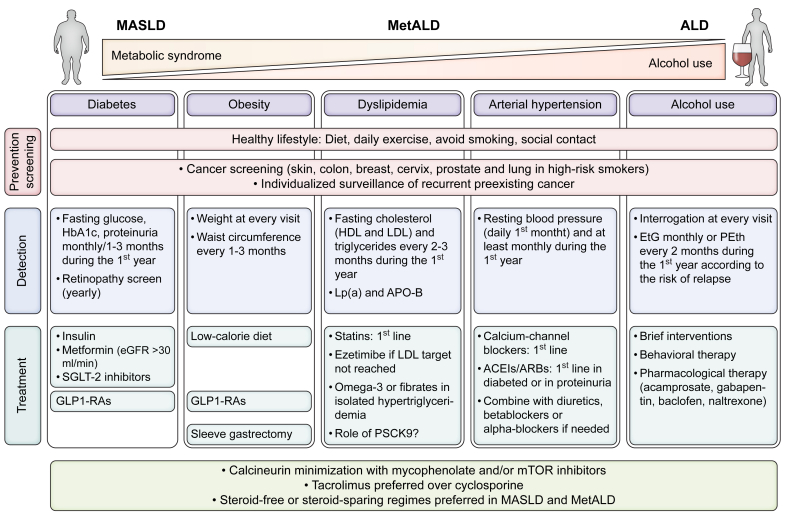
Recommendations for the management of comorbidities after LT in patients with MASLD, MetALD and ALD. ACEI, angiotensin converting enzyme-inhibitor; ALD, alcohol-related liver disease; ARBs, angiotensin receptor blockers; AUD, alcohol use disorder; eGFR, estimated glomerular filtration rate; EtG, ethyl glucuronide; GLP1-RAs, glucagon-like peptide-1 receptor agonists; LT, liver transplantation; MASLD, metabolic dysfunction-associated steatotic liver disease; MetALD, MASLD with increased alcohol intake; mTOR, mammalian target of rapamycin; PEth, phosphatidylethanol; SGLT2, sodium-glucose cotransporter-2.

**Fig. 4:**
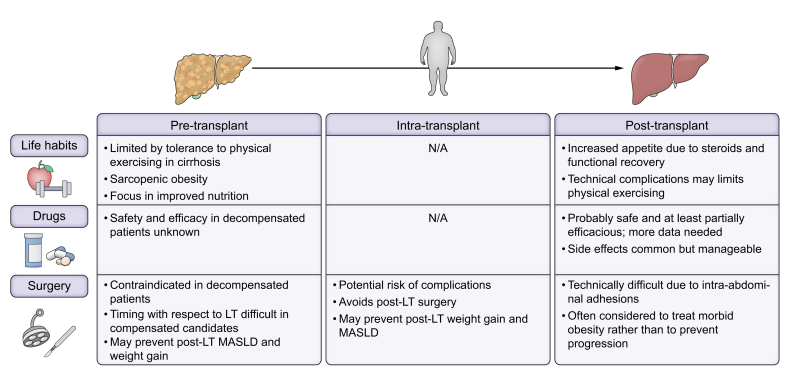
Main challenges in the management and treatment of obesity before and after LT. LT, liver transplantation; MASLD, metabolic dysfunction-associated steatotic liver disease.

Obesity is a central component of MS and is present in most patients with MASLD.^63^ In addition, recent data show that the number of LT candidates with obesity is increasing.^64^ While the association between obesity and post-LT survival is controversial, the impact of obesity on early post-transplant complications (particularly in patients with surgical complexity due to previous abdominal surgery or complete portal thrombosis) and post-LT MS is well established.^65^ Management of obesity before LT is complex and should be performed by a multidisciplinary team, including dietary and lifestyle counselling, anti-obesity drugs and the consideration of surgery.^65^^,^^66^ A recent report showed that a multidisciplinary programme incorporating dietary, pharmacologic, and psychological support led to significant weight loss in LT candidates, allowing transplantation in patients previously excluded due to obesity.^67^ Challenges to obesity treatment before LT arise from liver dysfunction and portal hypertension, which limit the efficacy of physical exercise and lifestyle interventions and affect the safety of pharmacological and surgical treatments (Fig. 3). Endoscopic bariatric treatment may be considered before LT, but adverse events including decompensation can occur.^68^ Another potential approach for morbid obese candidates is bariatric surgery. Sleeve gastrectomy before LT is currently only recommended in candidates with morbid obesity and Child-Pugh class A compensated cirrhosis without clinically significant portal hypertension.^16^ A very recent multicentre study has shown that simultaneous LT and sleeve gastrectomy leads to less post-LT diabetes, arterial hypertension and graft steatosis, and induces higher weight loss than LT alone in LT recipients with BMI >30.^69^ Although this report did not show an increased risk of complications or mortality, another recent study has reported unacceptably high post-LT mortality, highlighting the need for accurate patient selection and challenging the applicability of this approach.^70^ (Fig. 4).

#### Coronary artery disease

The prevalence of CAD in LT candidates has been reported to be 21-32%, and SLD, either MASLD or ALD, is a risk factor for CAD.[71], [72], [73] Despite not being supported by prospective data, most guidelines recommend considering percutaneous coronary intervention (PCI) of asymptomatic severe coronary lesions in LT candidates^16^^,^[74], [75], [76], [77] due to the poor outcomes of these patients when undergoing LT with untreated severe CAD.^78^^,^^79^ Using this approach, LT candidates with treated asymptomatic significant CAD do not appear to have higher mortality than patients without CAD,^80^^,^^81^ a finding that may support the efficacy of treatment in equalising outcomes with those of patients with less severe (or no) CAD. Though infrequent, severe complications of PCI can occur,^81^ and the indication for PCI needs to consider the severity of CAD and the coronary territory at risk, and the patient’s liver function, clinical status and risk of bleeding.

Before considering treatment, CAD must be diagnosed. Recent EASL guidelines,^16^ an AHA-AST scientific statement^74^ and several expert reviews[75], [76], [77] recommend anatomic tests – mainly CT coronary angiography with coronary artery calcification score (CACS) – in patients at risk of CAD. While these tests are highly sensitive and consequently are very useful to rule out the presence of significant CAD, specificity is around 30-40%, leading to false positive results and more invasive coronary angiographies than strictly necessary.^73^ The indication to evaluate the presence of CAD and perform CT coronary angiography with CACS is based on the pre-test consideration of risk, which mainly depends on the presence of CMRFs. Most centres include MASLD as an independent risk factor to indicate the evaluation of CAD. Using LT-specific algorithms like the *CAD-LT* score^82^ may help refine our selection of candidates needing CAD assessment.

### MetALD

Patients with MetALD represent a heterogeneous group in whom the relative contribution of alcohol intake and the severity of metabolic dysfunction can vary widely. Many individuals discontinue alcohol use even before evaluation and may not fulfil criteria for AUD. Nonetheless, characterisation of alcohol consumption by addiction specialists – from low-risk consumption to established AUD – is essential. Simultaneously it is also key to address the components of the MS, since the metabolic phenotype may also change in end-stage liver disease. Importantly, recent data from the United Network for Organ Sharing show that patients with MetALD have a higher risk of waiting list removal than those with ALD.^20^ In conclusion, LT assessment of patients with MetALD should integrate both the general considerations of MASLD and ALD and the specific aspects of AUD, as summarised in Table 2. This comprehensive approach not only ensures adequate patient assessment but also supports lifestyle modifications that are critical for long-term outcomes.

## Management of SLD after LT

### Post-transplant management in ALD recipients

After LT, patients with ALD demonstrate survival rates that are at least comparable – and in some studies even superior – to those observed in patients transplanted for HCC or viral hepatitis (5-year and 10-year patient survival: 75-81% and 60-63%, respectively).^83^ However, beyond the 5-year mark, both patient and graft survival may be compromised by long-term complications. The main drivers of morbidity and mortality after LT for ALD include cardiovascular events, development of MS, depression, and malignancies rather than disease recurrence.[84], [85], [86], [87] Due to the high risk of *de novo* malignancies after LT,^74^ it is essential to minimise exposure to calcineurin inhibitors and to implement enhanced cancer surveillance beyond standard population-based screening, including lung cancer screening for high-risk patients with low-density thorax CT (cumulative dose >20 pack-years, active smokers or those who quit <15 years ago^85^). This is particularly important for detecting aerodigestive tract cancers (head, neck, and oesophagus), lung cancer and skin cancer^88^^,^^89^ whose incidence after LT is higher than for other aetiologies of liver disease,^90^ owing to the concomitant exposure to alcohol and tobacco. In patients with abnormal liver function tests or known or suspected alcohol relapse, ultrasonography and elastography are useful non-invasive tools to screen for hepatic steatosis. However, liver biopsy remains the gold standard for assessing the presence of inflammation, fibrosis, and possible rejection.^16^

In the medium and long term, patients transplanted for SAH or for ALD without SAH do not seem to have different outcomes, with survival rates of approximately 80% at 2-3 years.^39^^,^^40^ The benefit brought by LT at 2-3 years does not seem to be different in patients transplanted with or without a sobriety period^32^^,^^33^^,^^40^ and a modelling study in patients with SAH has observed that the best gain of survival was obtained in the case of immediate listing compared to delayed LT.^91^ The negative impact of alcohol recurrence in patients transplanted for ALD has been demonstrated in many studies, showing an increased risk of graft loss related to rejection and recurrence of cirrhosis.^28^^,^^92^ However, it is important to emphasise that the harmful impact of alcohol recurrence is not significant during the first 5 years after LT – which is considered the benchmark time frame for evaluating long-term transplant outcomes – and generally becomes evident only beyond this period.^93^

#### Detection and management of alcohol relapse after LT

The risk of alcohol recurrence has long been underestimated, largely because of the lack of reliable methods to measure alcohol consumption after LT. The medical interview should remain the gold standard for assessing a patient’s alcohol consumption and, when necessary, initiating appropriate management for AUD. However, alcohol relapse after LT can often go unnoticed by attending physicians, especially in the absence of overt clinical signs or laboratory abnormalities.^28^ Besides the “yes/no” interview, several tools have been specifically designed by specialists in addiction to capture actual alcohol consumption, the most accurate being the TLFB (timeline follow back) calendar.^94^ This tool is semi structured and reliable but has the disadvantage of being retrospective and prone to memory biases. The short AUDIT-C (alcohol use disorders identification test – consumption) can also be used to facilitate the medical interview, which was shown to be better conducted by a specialist in addiction than by a hepatologist in a French prospective single-centre study.^95^ Similarly, the presence of a multidisciplinary team following the patient with ALD after LT, including specialists in addiction within the transplantation centre, has been proven to decrease the risk of alcohol recurrence after LT, thereby decreasing the risk of rejection and mortality after LT.^35^^,^^96^

Since patients may be unwilling to report alcohol use or even choose to hide alcohol use, detecting relapse after LT can now be supported by direct biomarkers (Fig. 3). The two most widely used are ethylglucuronide (ETG), detected in urine or hair, and phosphatidylethanol (PEth), measured in whole blood. A German study found urinary ETG to be the best predictor of relapse,^97^ though it is only detectable for 72–96 h. Hair ETG offers a longer window (up to 6 months) but is less accessible.^98^ PEth, increasingly available, reflects intake over 3–4 weeks and has shown high sensitivity and specificity (90–100%).[99], [100], [101] However, accuracy varies by concentration, with high values being most reliable,^99^ and levels can be influenced by factors like BMI and haemoglobin.^102^

Due to varying definitions and populations, the reported risk of alcohol relapse after LT in patients with AUD is highly variable. A systematic review published in 2001 before the widespread use of modern biomarkers observed a risk of recurrence of any alcohol consumption of approximately 30% at 7 years after LT.^103^ More recently, EASL reports in their guidelines a risk of any relapse of 25-35% at 2 years, and a risk of sustained alcohol recurrence of 20% at 3 years.^16^ In patients with SAH, the large American consortium ACCELERATE (including data on 147 patients) showed evidence of any alcohol use after LT in 28% of patients surviving to home discharge.^42^ The prospective study QuickTrans observed, as recorded by TLFB, any alcohol consumption at 2 years in 35% of patients transplanted for AH, compared to 25% in patients with ALD and at least 6 months of abstinence.^40^ Although the difference was not statistically significant, heavy alcohol relapse was more frequent in SAH recipients.

In summary, patients with ALD have a good outcome after LT and a significant period of abstinence before placement on the waitlist is no longer mandatory. Conversely, the management after LT still needs to be improved, specially with respect to addressing addictions. An integrated team including addiction specialists, hepatologists, social workers, and nurses helps reduce alcohol recurrence and may lower mortality but many centres face significant resource limitations (*i.e.* insufficient health professionals trained in addiction management).^35^^,^^104^

### Post-transplant management in MASLD recipients

Patients with MASLD also have comparable survival rates to those transplanted for other end-stage liver diseases (5- and 10-year patient survival: 75-83% and 60-69%, respectively). The main causes of mortality in patients with MASLD who have a functioning graft are cardiovascular disease complications and cancer.^24^ LT recipients with prior MASLD are at higher risk of developing *de novo* pancreatic and colon cancer than those transplanted for other aetiologies,^105^ while MASLD represents an additive factor for any *de novo* malignancy.^106^ Therefore, strict adherence to carcer surveillance strategies is necessary for these patients and more studies are warranted to determine whether patients with MASLD require individualised cancer screening programmes.

Recurrence of steatosis after LT has been reported in up to 80% of patients at 5 years, while MASH recurrence has been reported in 28-60% of patients[107], [108], [109], [110] (Table 3). Corticosteroids, calcineurin inhibitors and mTOR inhibitors may contribute to post-transplant steatosis by promoting insulin resistance, hypertension and dyslipidaemia and increasing hepatic lipid accumulation.^111^ Data regarding the incidence of progressive fibrosis or graft cirrhosis is conflicting; and despite the high recurrence rate, the impact of recurrent MASLD on graft survival does not seem to be particularly significant, maybe due to the competing cardiovascular risk that accompanies MASLD recurrence. It should be noted, however, that studies on MASLD recurrence are limited by the inclusion bias derived from the different strategies used to monitor and diagnose recurrence.

**Table 3:** Prospective studies assessing the incidence of MASLD after LT.

Authors (Ref.) country	N	Follow-up or median time post-LT (months)	Diagnostic modality	Prevalence or incidence (%)	Risk factors and other relevant information
Villeret *et al.*^110^ France	150	56	Biopsy	MASLD 85% MASH 60%	Fibrosis ≥F2 at 5 years: 48%. F3/4 at 5 years: 20%.
Gitto *et al.*^112^ Italy	194	55	Biopsy	20% *de novo*	BMI, diabetes, metabolic syndrome
Charlton *et al.*^113^ USA	546	12	Biopsy	75%	Not reported
Kim *et al.*, South Korea^114^	32	13	Biopsy	28%	PNPLA3
Dobrindt *et al.*,^115^ Germany	414	12-60	Biopsy (n = 271)	43% MASLD at 5 years 23% MASH at 5 years	*De novo* diabetes, liver fibrosis at 5 years: 43% in those with diabetes
Adali *et al.*^116^ Turkey	128	66	Transient elastograhy + CAP	34% overall (12% recurrent; 22% *denovo*)	Diabetes
Choudhary *et al.*^117^ India	117	62	Ultrasonography	48% overall (39% recurrent;31% *de novo*)	BMI, diabetes, dyslipidaemia
Mikolasevic *et al.*^118^ Croatia	175	48	Transient elastograhy	69%	Diabetes, obesity
Eshraghian *et al.*^41^ Iran	178	41	Ultrasonography + transient elastograhy	44%	HOMA-IR, diabetes, adiponectin, leptin
Andrade *et al.*^119^ Brazil	119	13	Ultrasonography	43% *de novo* MASLD 56% recurrent MASLD	HDL-cholesterol

LT, liver transplantation; MASH, metabolic dysfunction-associated steatohepatitis; MASLD, metabolic dysfunction-associated steatotic liver disease.

#### Detection and management of MASLD after LT

There is no widely accepted strategy to monitor and detect MASLD after LT. Considering cost-efficacy concerns and limitations of serological scores in LT recipients,^120^^,^^121^ an initial evaluation with ultrasound and transient elastography (TE) with controlled attenuated parameter (CAP) in the first 6-12 months after LT, followed by monitoring with TE and CAP biannually, may be a reasonable approach in asymptomatic MASLD LT recipients with normal liver tests. Unlike non-transplanted patients, LT recipients have multiple potential causes of abnormal liver tests (vascular, biliary, infectious, *etc*.); therefore, systematic evaluation of alternative aetiologies and a low threshold for liver biopsy are essential in this population. Once MASLD recurrence has been diagnosed, monitoring these patients with TE and CAP could be useful, provided liver function tests show no significant changes. It should be noted that no validated strategy currently exists to monitor recurrent MASLD.

Risk factors for MASLD recurrence include pre- and post-LT obesity and CMRFs and *PNPLA3* polymorphisms.[107], [108], [109], [110]^,^^120^^,^^122^ Regarding immunosuppression, a meta-analysis found that only sirolimus use was associated with recurrent MASLD,^107^ although this may be confounded by the indications for its use. No clinical donor-related factors have been associated with the risk of recurrent MASLD, and the impact of donor *PNPLA3* polymorphisms is controversial.^122^^,^^123^ Current treatment of post-LT MASLD is limited to the management of CMRFs,^124^ particularly obesity. There are no data regarding the use of specific MASLD (not-obesity related) drugs like resmetirom, the safety and efficacy of which needs to be determined in this population.

#### Management of cardiometabolic risk factors in MASLD recipients

Cardiovascular disease is one of the leading causes of long-term death after LT,^125^ and MASLD has been shown to be a risk factor for cardiovascular events in some,^126^ but not all, studies.^127^ Post-LT MS and kidney dysfunction are also associated with an increased risk of post-LT cardiovascular events.^128^ Managing CMRFs after LT is complex and requires multidisciplinary teams, which may improve the control of these factors.^128^^,^^129^ While a detailed review of the management of CMRF after LT is beyond the scope of this review, Fig. 3 summarises the main recommendations for post-LT treatment of arterial hypertension, diabetes, dyslipidaemia,^130^^,^^131^ and alcohol use. Obesity is particularly frequent in MASLD LT recipients and drives the progression of MASLD recurrence and the grade of control of the other CMRFs. Therefore, obesity is probably the most important modifiable condition to target in order to optimise outcomes in this population.^132^ A multidisciplinary approach that considers all potential therapies is mandatory in these patients.

The main pillar of obesity treatment is lifestyle interventions. Nonetheless, interventions like routine medical counselling have limited effectiveness in preventing weight gain after LT,^133^ and most patients may require pharmacologic therapy. The high efficacy of glucagon-like peptide-1 receptor agonists (GLP-1RAs) in the treatment of obesity in the general population has prompted their use in LT recipients. However, there are still few studies reporting their safety and efficacy after LT. Very recently, Yakubu *et al.*^134^ compared 38 LT recipients with diabetes treated with GLP1-RAs with 38 LT recipients with diabetes treated with insulin. GLP1-RA-treated patients lost 8% of body weight, compared with a 10% weight gain in insulin-treated patients, and they presented a significant decrease in CAP values. Importantly, no safety issues or graft dysfunction were reported. Similar results have been shown in other small series.[135], [136], [137] Overall, these results suggest that GLP-1RAs may be used as a first-line treatment of obesity in LT recipients, although long-term data are still needed. There are no data regarding the usefulness or safety of dual agonists of GLP-1 and glucose-dependent insulinotropic polypeptide receptors like tirzepatide in this population. In LT recipients unresponsive to medical therapy, bariatric surgery – preferably sleeve gastrectomy to avoid immunosuppressant malabsorption and preserve biliary access – may be effective for weight loss and CMRF control.^138^ However, prior surgical adhesions limit feasibility due to increased complication risk. No data exist on endoscopic bariatric therapy post-LT.

Finally, considering the prevalence of CMRF in this population, screening of cardiovascular disease may be considered. While there is no information regarding the best strategy to screen and prevent cardiovascular disease in LT recipients, general population recommendations should apply, including the assessment of risk using validated algorithms such as the PCE (pooled cohort equations) or the SCORE (systematic coronary risk evaluation), and appropriate treatment of CMRFs as shown in Fig. 3. Interestingly, both American and European guidelines^139^^,^^140^ support a role for CACS in some subpopulations to refine risk stratification and treatment intensity, particularly for statins. As mentioned above, a significant number of these patients have a CACS measured before LT, which may consequently be used for this purpose. The usefulness of post-LT CACS to improve cardiovascular disease risk management is unknown.

### Post-transplant management in MetALD recipients

Patients with MetALD should be managed according to the established recommendations for MASLD and ALD, with interventions tailored to their predominant phenotype. A multidisciplinary approach is essential to address the overlapping pathophysiological mechanisms and clinical consequences of MetALD. The main goals mirror those in MASLD and ALD: promoting and maintaining a healthy lifestyle, ensuring early detection of alcohol use, preventing and treating both alcohol relapse and CMRFs, and enabling early detection of graft dysfunction, cardiovascular complications, and cancer.

## Steatotic liver disease in liver donors

The increasing prevalence of hepatic steatosis among potential donors poses a growing challenge in LT. SLD in the donor increases the risk of graft failure and disproportionately decreases the chances of utilising many donors.^141^ Steatotic grafts are more vulnerable to ischaemia-reperfusion injury (IRI), causing mitochondrial and microvascular damage and Kupffer cell activation.^142^ While mild steatosis is usually well-tolerated, grafts with >60% macrosteatosis are contraindicated for transplant with static cold storage (SCS).^143^ Those with additional risk factors or >30% steatosis require individualised assessment^144^ and steatosis >10% is avoided in living donor LT.^145^ Donation after circulatory death (DCD) livers with steatosis are especially prone to IRI, post-reperfusion syndrome, primary graft non-function, and early-allograft dysfunction (EAD). Evaluating donor steatosis is complex; although biopsy remains the gold standard, its use in deceased donors is limited by variability and logistics.^146^ The Banff Working Group introduced standardised histological categories, improving consistency in classification,^147^ and the utilisation of AI may enable unbiased automated evaluation of steatosis.^148^ Clinically, mild steatosis (<30%) is generally acceptable, whereas moderate to severe steatosis (>30%) requires risk evaluation and mitigation.^143^^,^^146^ MRI-proton density fat fraction offers a reliable, non-invasive alternative for steatosis assessment; however, it is not routinely performed in clinical practice.^149^

Machine perfusion strategies offer a promising path to improve outcomes and enable safe use of steatotic grafts.[150], [151], [152] Hypothermic perfusion aids preservation and mitochondrial repair, while normothermic perfusion allows for real-time viability assessment and supports hepatic function.^152^ In DCD donors, *in situ* normothermic regional perfusion helps restore oxygen delivery and assess graft viability, preventing microcirculatory thrombosis.^150^ *Ex situ* normothermic machine perfusion (NMP) maintains the liver at 37 °C with oxygenated blood, reversing metabolic damage and enabling use of previously discarded grafts.^153^ NMP also reduces hepatic fat via metabolic activation and β-oxidation, as shown in a Zurich study extending perfusion up to 12 days with tailored nutrition regimens.^154^ Ischaemia-free techniques further broaden the use of high-risk steatotic grafts by eliminating ischaemic injury.^155^ Additionally, hypothermic oxygenated perfusion (HOPE) lowers graft temperature to 10–12 °C while delivering oxygen, mitigating IRI, particularly in DCD livers. Importantly, unlike HOPE, NMP is able to functionally assess organ viability.

Dynamic liver preservation techniques like normothermic and hypothermic perfusion have improved the safety of marginal organ transplantation compared to SCS,^144^^,^[156], [157], [158] as shown in Table 4. Both HOPE and NMP consistently reduce EAD, a marker of IRI.^159^ A network meta-analysis confirmed HOPE’s ability to lower EAD risk (relative risk [RR] 0.53) and enhance 1-year graft survival (RR 1.07), even with prolonged warm ischaemia.^157^^,^^160^^,^^161^ NMP also offers improved haemodynamic stability and leads to fewer cases of post-reperfusion syndrome, which is further reduced with NMP plus ischaemia-free LT (RR 0.49 and 0.15, respectively).^159^^,^^162^ Biliary complications, especially non-anastomotic biliary strictures (NAS), remain concerns in DCD and steatotic grafts. Both HOPE and abdominal normothermic regional perfusion significantly lower NAS rates.^157^^,^^163^ In a real-world cohort of 1,202 HOPE-perfused grafts, NAS incidence was 2.5% in DBD and 11.5% in DCD grafts, with >90% 5-year graft survival.^164^ NMP and SCS are associated with similar needs for renal support, intensive care stay, and hospitalisation length.^162^ In conclusion, the integration of refined histopathological assessment criteria, innovative machine perfusion technologies, and metabolic modulation strategies offers a transformative opportunity to expand the donor pool with steatotic livers.

**Table 4:** Current evidence on the effects of *in situ* and *ex situ* machine perfusion in steatotic and non-steatotic liver grafts.

	*In situ* perfusion†	*Ex situ* perfusion∗	
	Regional normothermic perfusion (NRP)	Normothermic machine perfusion (NMP)	Hypothermic machine perfusion (HOPE)∗
Reperfusion syndrome	Reduced risk^165^	Reduced risk^151^^,^^159^^,^^162^^,^^165^	Reduced risk^157^^,^^166^
Early allograft dysfunction	Reduced risk^167^	Reduced risk^159^^,^^168^	Reduced risk especially in steatotic grafts^157^^,^^164^^,^^166^^,^^169^
Anastomotic biliary complications	Reduced risk^150^^,^^156^^,^^167^	Reduced risk	Reduced risk^157^
Non-anastomotic biliary complications	Reduced risk^156^^,^^167^^,^^170^	Decreased risk,^168^ especially in DCD and/or steatotic grafts	Reduced risk^171^
Rejection	-	Possible reduction^159^^,^^172^	Possible reduction^159^^,^^172^
Hospital stay	-	Reduced in DCD^173^	Possibly reduced^166^
Graft loss	Similar or reduced risk in DCD donors^150^^,^^156^	Reduced risk in DCD donors^162^^,^^173^	Reduced risk^169^
Patient survival	Increased survival in DCD donors^171^	Increased survival in DCD donors^173^	Unaffected despite using marginal donors^164^
Rescue discarded donors	-	Increased use of DCD donors^153^^,^^168^	Increased use of marginal donors^152^
Complication comprehensive index	Reduced^171^	Reduction of complications and readmission rate in DCD donors^155^^,^^173^	Reduction in major complications^163^^,^^169^

DCD, donation after circulatory death.

† Compared to rapid extraction in DCD.

∗ Compared to static cold storage.

## Research gaps and future directions

• Lack of approved pharmacotherapies to reduce steatosis in candidates with MASLD and patients with *de novo* MASLD after LT. Limited data on the safety and efficacy of emerging agents (*e.g.* GLP-1RAs, SGLT2 inhibitors, resmetirom) in decompensated cirrhosis. Lack of studies evaluating MASLD recurrence treatment (*e.g.* resmetirom, lanifibranor) in LT recipients and the safety and efficacy of endoscopic or pharmacological obesity interventions in LT recipients remain unknown.
• Most LT series in SAH patients exclude individuals with a history of prior hepatic decompensation. Nonetheless, a study evaluating LT outcomes in a selected cohort of SAH patients with prior decompensation found a significantly higher adjusted post-LT mortality (adjusted hazard ratio 2.72; 95% CI 1.61–4.59) and increased risk of harmful alcohol use (adjusted hazard ratio 1.77; 95% CI 1.07–2.94). Despite these risks, the 3-year survival was comparable to that of patients transplanted for other accepted indications, suggesting a survival benefit in this subgroup and underscoring the need for larger studies to refine selection criteria.^47^
• Several questions remain to be answered in the field of machine perfusion in steatotic donors, including optimising the perfusion duration, overall protocols, targeted pharmacologic interventions during perfusion and multimodal perfusion strategies with HOPE and NMP.

## Concluding remarks

SLD is increasing in prevalence worldwide, leading to an increased number of LT candidates and liver donors with SLD. The selection of candidates with SLD should include accurate assessment of alcohol use along with metabolic and cardiovascular comorbidities. SLD after LT can result in metabolic complications (obesity, diabetes, dyslipidaemia, hypertension) and alcohol relapse, graft-related complications (steatosis, steatohepatitis, fibrosis), and long-term risks (cardiovascular disease, cancer, and alcohol relapse). Innovative management strategies include: a) personalised immunosuppression (calcineurin-inhibitor minimisation, steroid avoidance); b) early detection of alcohol use (medical interview and biomarkers), *de novo* SLD (ultrasonography, TE, biopsy when needed), cancer (screening protocols), and CMRFs; c) targeted interventions for obesity (GLP-1RAs, SGLT2 inhibitors, bariatric surgery), CMRFs, and AUD (psychological therapy, pharmacological treatment, and social support). Multidisciplinary management is essential to address all these aspects effectively.

## Abbreviations

ACLF, acute-on-chronic liver failure; ALD, alcohol-related liver disease; AUD, alcohol use disorder; CACS, coronary artery calcification score; CAD, coronary artery disease; CAP, controlled attenuated parameter; CMRF, cardiometabolic risk factors; DCD, donation after circulatory death; EAD, early allograft dysfunction; GLP-1RAs, glucagon-like peptide-1 receptor agonists; HCC, hepatocellular carcinoma; HOPE, hypothermic oxygenated perfusion; LT, liver transplantation; MASLD, metabolic dysfunction-associated steatotic liver disease; MELD, model for end-stage liver disease; MetALD, MASLD with increased alcohol intake; MS, metabolic syndrome; NAS, non-anastomotic biliary strictures; NMP, normothermic machine perfusion; NRP, normothermic regional perfusion; PEth, phosphatidylethanol; RR, relative risk; SAH, severe alcohol-associated hepatitis; SCS, static cold storage; SLD, steatotic liver disease; TE, transient elastography.

## Financial support

This work was partially supported by Beca Fundación Mutua Madrileña 2022 (AP180672022) and by the Instituto de Salud Carlos III (ISCIII) and co-funded by the European Union through project PI22/01234. JC and GC are supported by AGAUR (Departament de Recerca i Universitats de la Generalitat de Catalunya, exp. 2021-SGR-01331).

## Authors contributions

JC and AL conceived the study and designed the manuscript. JC, AL, GC, YF and LCNW drafted the manuscript. JC and GC designed the figures. JC and AL revised the manuscript. All authors read and approved the final manuscript.

## Declaration of generative AI and AI-assisted technologies in the writing process

During the preparation of this work the author(s) used ChatGPT, Gemini and Google AI studio to create images and English edition/revision. After using this tool/service, the authors reviewed and edited the content as needed and take full responsibility for the content of the publication.

## Conflict of interest

The authors declare that they have no conflict of interest and no affiliations with or involvement with any organization or entity that has financial interest in the subject matter or materials discussed in this manuscript.

Please refer to the accompanying ICMJE disclosure forms for further details.
